# Uranine.—A New Aniline Color

**Published:** 1879-01

**Authors:** 


*Uranine—A New Aniline Color.*—We have received, with the compliments of the *Scientific American*, a specimen of the newest, and what is claimed to be the most remarkable, of all the aniline group of coloring substances, all of which are extracts from coal tar. This color is called "uranine;" it produces one of the most brilliant and translucent shades of emerald greens which it is possible to conceive of, and is said by the chemists to be the most highly fluorescent body known to science. Its coloring power and diffusiveness are so great that it is claimed that a single grain of the powder will impart a marked tinge to eleven barrels or 350 gallons of water. A few atoms of it sprinkled upon the surface of a tumbler of water very soon fills the glass with what appear to be fresh and delicate rootlets of moss of the most vivid yet delicate green. In some lights the hues have a peculiar pearly lustre and translucency, and many of the transient lights of water tinged with it have all the evanescent brilliancy and variety of color of the opal. This new color, the proper mordant for which is said to be glycerine, promises to become one of the most important and valuable dyes known in the arts.

